# Customizable Fully Structured Vector Trajectory Beams in the Focal Plane via a Single‐Layer Metasurface

**DOI:** 10.1002/nap2.70141

**Published:** 2026-05-24

**Authors:** Zhibang Tan, Zebin Chen, Zepeng Chen, Yantao Zheng, Min Ouyang, Wei Hu, Qing Wang, Lijun Wu, Qian Shou

**Affiliations:** ^1^ Guangdong Provincial Key Laboratory of Nanophotonic Functional Materials and Devices, School of Optoelectronic Science and Engineering South China Normal University Guangzhou China; ^2^ Institute of Nonlinear Optics, College of Science JiuJiang University Jiujiang China

**Keywords:** degrees of freedom, metasurfaces, Poincaré sphere, trajectory beams, vector vortex beams

## Abstract

As a class of structured light exhibiting unique spatial configurations, trajectory beams (TBs) feature the characteristic parameters of the light field, also referred to as the degrees of freedom (DoFs) of the light field, distributed along predefined trajectories. However, achieving TBs in which multiple DoFs are completely decoupled remains a significant challenge. Here, we demonstrate the generation of customizable fully structured vector trajectory beams (VTBs) in the focal plane using a single‐layer metasurface designed via computer‐generated holography. The controllable DoFs encompass the trajectory shape and the distributions of amplitude, phase, and polarization along the predefined trajectory. The generated TBs surpass existing methods in the number of controllable DoFs. More significantly, by shifting the key principle of the design from angular‐variant topological phases to spatial trajectory, they surpass conventional vortex beams based on the prior principle, including their more advanced derivatives. The demonstrated ability to independently control multiple DoFs holds great promise in enhancing the channel capacity in optical communication.

## Graphical abstract

This work introduces a unique class of structured light—trajectory beams—where all degrees of freedom evolve dynamically along the predefined trajectory. By employing computer‐generated holography, single‐layer metasurfaces achieve the complete decoupling of trajectory shape and the distributions of amplitude, phase, and polarization along the path, providing a highly flexible platform for multidimensional light‐field manipulation.

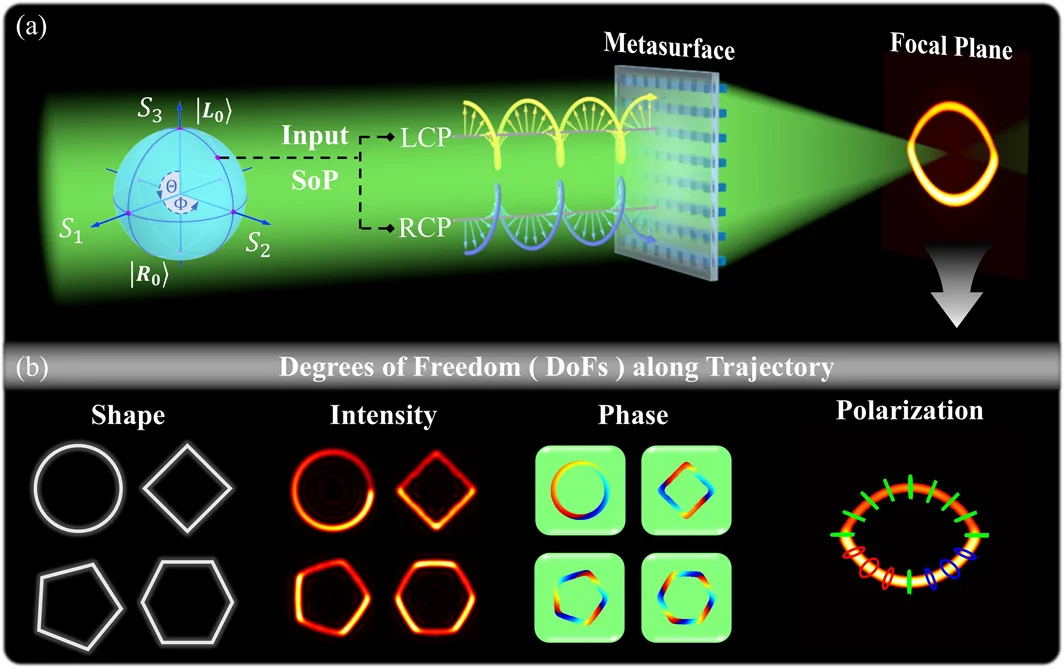

## 1 Introduction

By modulating degrees of freedom (DoFs) including amplitude, phase, and polarization, either singly or jointly, complex structured light fields with distinct physical properties can be generated. Representative examples such as vortex beams with helical phases [1], non‐diffracting Bessel beams [2], and vector beams with spatially dependent polarization [3], have revealed numerous novel physical phenomena. These fields are of great theoretical and practical significance in optical communication [4, 5], particle manipulation [6, 7, 8], optical sensing [9, 10], and computational imaging [11, 12].

In response to the increasingly complex control requirements in modern optics, metasurfaces with their sub‐wavelength scale control advantages have provided a more flexible and compact platform, leading to significant progress in the past decade [13, 14, 15, 16, 17]. As a focal point in light field manipulation, vortex beams have garnered significant attention due to their distinctive helical phase wavefronts and their ability to carry orbital angular momentum (OAM) [18, 19, 20]. In recent years, researchers have incorporated additional DoFs into vortex beams—such as amplitude and polarization—to generate advanced beam structures. These include generalized vortex beams that transcend traditional circular shape constraints [21], perfect vortex beams in which the topological charge is decoupled from the ring radius [22, 23], and vector vortex beams (VVBs) characterized by spatially non‐uniform polarization distributions [24, 25, 26]. Subsequent research has further dedicated efforts to the decoupled manipulation of multiple DoFs to construct even more complex structured light fields, with a typical example being generalized perfect vector vortex beams (GPVVBs), which simultaneously integrate the characteristics of the three aforementioned beam types [27, 28, 29, 30]. Although research on vortex beams has become relatively systematic and extensive, the core design principle of such beams fundamentally relies on an azimuthally varying phase gradient. Consequently, most existing studies still face significant limitations. On one hand, optical parameters such as phase inherently vary along the azimuthal direction independently of any predefined trajectory, and this decoupling between phase evolution and spatial paths severely restricts potential applications in specific scenarios. On the other hand, the DoFs in most existing beams remain constrained; the beam morphology is generally confined to circular or elliptical shapes, and the polarization ellipticity typically exhibits a uniform distribution.

Distinct from vortex beams, a class of structured light centering on the trajectory as the core manipulation object can effectively surmount the mentioned limitations [31, 32, 33, 34, 35]. Its core feature is that the amplitude, phase, and polarization of the light field vary along a predefined trajectory. We define these fields as trajectory beams (TBs). Recently, researchers have demonstrated the generation of beams with polarization varying along arbitrary spatial trajectories [36, 37]. However, for such TBs, the decoupling and independent control among multiple DoFs—including the trajectory shape and the distributions of amplitude, phase, and polarization along the trajectory—remain a research direction that urgently demands in‐depth exploration.

To address the mentioned challenges, this paper proposes customizable fully structured vector trajectory beams (VTBs) in the focal plane that can be directly generated via a single‐layer metasurface (Figure 1). Compared with the existing related studies (see Supporting Information S1: Table S1 in Supplementary Section 1 for details), our work has significant advantages, as follows: (1) It overcomes the current limited number of independently controllable DoFs, achieving full command over all spatial DoFs in the focal plane—including its shape and the distributions of amplitude, phase, and polarization; (2) moreover, the arbitrary control of polarization has been achieved, including ellipticity and orientation angle; and (3) crucially, all the control DoFs change along the trajectory, and this characteristic is beyond the reach of traditional beams constructed based on azimuthally varying vortex phases. This work enables the construction of complex highly adapted structured light fields tailored to specific application scenarios, holding significant theoretical importance and practical value in frontier domains such as precise particle manipulation, multi‐dimensional optical encryption, and customized optical communication.

**FIGURE 1:**
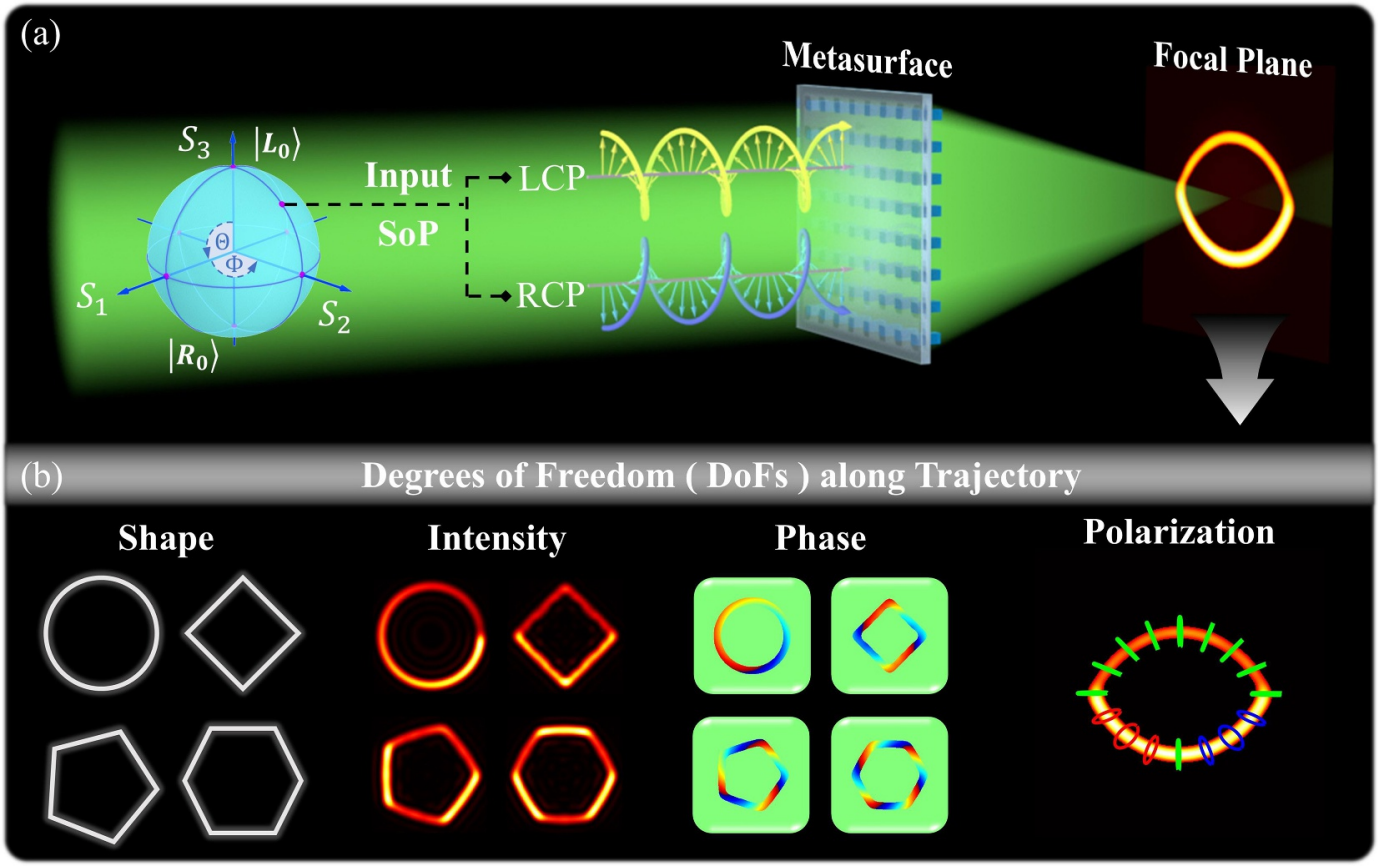
Schematic illustration of generating customizable fully structured VTBs in the focal plane via a single‐layer metasurface. (a) Upon illumination with incident light of a specific state of polarization (SoP) mapping to a defined coordinate (Θ,Φ) on the Poincaré sphere (PS), a customized VTB is generated in the focal plane. (b) DoFs: trajectory shape, and distributions of amplitude, phase, and polarization along the predefined trajectory. Green, red, and blue colors correspond to linear, left‐handed, and right‐handed polarization, respectively.

## 2 Design Principle

### 2.1 Mapping Relationship Between the Optical Field in the Focal Plane and the Hologram in the Incident Plane

The customizable fully structured VTBs are synthesized by coherent superposition of two orthogonal circularly polarized TBs, each capable of independent manipulation across multiple DoFs. Therefore, we first address the generation principle of a single‐channel TB. The hologram required for the metasurface is obtained via the inverse discrete Fourier transform (IDFT) of discrete point sources sampled along the target trajectory in the focal plane [35, 38, 39], combining the phase profile of a Fourier lens. The mapping relationship between the optical field in the focal plane and the hologram in the incident plane can be described as follows:

(1) $$\begin{array}{c} H_{L,R}\left(x_{j},y_{j}\right)=\sum_{n=1}^{N}E_{L,R}\left(x_{n},y_{n}\right)\exp\left[i\frac{2\pi }{\lambda f}\left(x_{j}x_{n}+y_{j}y_{n}\right)\right]\exp\left[i\varphi_{\text{lens}}\left(x_{j},y_{j}\right)\right], \end{array}$$

here $E_{L,R}\left(x_{n},y_{n}\right)=A_{L,R}\left(x_{n},y_{n}\right)e^{i\varphi_{L,R}\left(x_{n},y_{n}\right)}$ denotes the complex amplitude of the nth point $\xi_{n}\left(x_{n},y_{n}\right)$ along the target trajectory of the left circularly polarized (LCP) and right circularly polarized (RCP) channels in the focal plane, respectively. The focal spatial coordinates $\left(x_{n},y_{n}\right)$ are determined by the cumulative arc length $\xi_{n}=S\times \frac{n-1}{N-1}$ based on the principle of uniform arc length sampling. $S=\int_{\theta_{\text{start}}}^{\theta_{\text{end}}}\sqrt{{\left(\frac{dx}{d\theta }\right)}^{2}+{\left(\frac{dy}{d\theta }\right)}^{2}}d\theta$ represents the total arc length, N is the total number of discrete points, and $\left(x_{j},y_{j}\right)$ denote the coordinates of the hologram. $\varphi_{\text{lens}}\left(x_{j},y_{j}\right)=-\frac{2\pi }{\lambda }\left(\sqrt{{\left(x_{j}\right)}^{2}+{\left(y_{j}\right)}^{2}+f^{2}}-f\right)$ represents the Fourier lens phase.

### 2.2 Mathematical Construction and Characterization of VTBs

VTBs possess complex states of polarization (SoPs). The composition of these SoPs typically arises from the superposition of two orthogonal polarization basis vectors carrying different vortices. VVB is a representative example of such beams with such complex SoPs distributions [40, 41]:

(2) $$\begin{array}{c} |E\left(\vartheta \right)\rangle =\cos\left(\frac{\Theta }{2}\right)e^{-i\frac{\Phi }{2}}e^{i\varphi_{L}\left(\vartheta \right)}|L\rangle +\sin\left(\frac{\Theta }{2}\right)e^{i\frac{\Phi }{2}}e^{i\varphi_{R}\left(\vartheta \right)}|R\rangle \end{array}$$

here Θ and Φ are two constant values. $\varphi_{L}\left(\vartheta \right)=l_{L}\vartheta$ and $\varphi_{R}\left(\vartheta \right)=l_{R}\vartheta$ denote the vortex phases of the respective components, where $l_{L}$ and $l_{R}$ represent the corresponding topological charges, and ϑ is the azimuthal angle. $|L\rangle =\frac{1}{\sqrt{2}}\left(e_{x}+ie_{y}\right)$ and $|R\rangle =\frac{1}{\sqrt{2}}\left(e_{x}-ie_{y}\right)$ constitute the pair of orthogonal circular polarization basis vectors. Because the vortex phase varies strictly along the azimuthal direction, the polarization distribution of the VVBs inherently varies along the azimuthal direction.

In this work, we overcome the inherent azimuthal dependence of conventional VVBs to achieve trajectory‐based control of the light field. This enables the on‐demand customized distributions of amplitude, phase, and polarization along the predefined trajectory. Accordingly, Equation (2) can be generalized as follows:

(3) $$\begin{array}{c} |E\left(\xi \right)\rangle =\cos\left(\frac{\Theta }{2}\right)e^{-i\frac{\Phi }{2}}A_{L}\left(\xi \right)e^{i\varphi_{L}\left(\xi \right)}|L\rangle +\sin\left(\frac{\Theta }{2}\right)e^{i\frac{\Phi }{2}}A_{R}\left(\xi \right)e^{i\varphi_{R}\left(\xi \right)}|R\rangle , \end{array}$$

here (Θ,Φ) serves as a global modulation factor corresponding to the incident polarization, enabling control over the overall polarization characteristics of the beam. In contrast, $A_{L}\left(\xi \right),A_{R}\left(\xi \right),\varphi_{L}\left(\xi \right),\varphi_{R}\left(\xi \right)$ act as local modulation factors along the predefined trajectory, allowing for the independent manipulation of the local amplitude and phase. This approach of arc length parameterization endows the system with extensive DoFs for trajectory‐based manipulation; theoretically, it supports the arbitrary trajectory shape and the distributions of amplitude, phase, and polarization along the predefined trajectory.

Furthermore, we incorporate a vortex phase into the phase design of Equation (3). Inspired by the concept of classical topological charge, we propose a generalized topological charge l, representing a continuous phase variation of 2πl along the predefined trajectory. Consequently, the phase components for the LCP and RCP can be described as: $\varphi_{L}\left(\xi \right)=l_{L}\left(2\pi \xi /S\right)$, $\varphi_{R}\left(\xi \right)=l_{R}\left(2\pi \xi /S\right)$. To elucidate the intrinsic connection between the phase and the polarization of the VTBs, and drawing an analogy to the definitions of phase order and polarization order in conventional vortex beams [42], we define a generalized phase order $l_{G}=\left(l_{L}+l_{R}\right)/2$ and a generalized polarization order $l_{P}=\left(l_{L}-l_{R}\right)/2$. Specifically, $l_{G}$ and $l_{P}$ represent the global phase and polarization orientation evolution along the predefined trajectory, which undergo total accumulations of $2\pi l_{G}$ and $2\pi l_{P}$, respectively. Incorporating Equation (3), the light field distribution can be derived as follows:

(4) $$\begin{array}{c} |E\left(\xi \right)\rangle =A\left(\xi \right)e^{i\varphi \left(\xi \right)}|\Psi \left(\xi \right)\rangle , \end{array}$$

where

(5) $$\begin{array}{c} A\left(\xi \right)=A_{L}\left(\xi \right)\cos\left(\Theta /2\right)\sqrt{1+\rho^{2}} \end{array},$$

(6) $$\begin{array}{c} \varphi \left(\xi \right)=l_{G}\frac{2\pi \xi }{S} \end{array},$$

(7) $$\begin{array}{c} |\Psi \left(\xi \right)\rangle =\frac{1}{\sqrt{2\left(1+\rho^{2}\right)}}R\left(l_{P}\frac{2\pi \xi }{S}-\frac{\Phi }{2}\right)\left[\begin{array}{c} 1+\rho \\ i\left(1-\rho \right) \end{array}\right] \end{array},$$

here A(ξ), φ(ξ), and |Ψ(ξ)〉 denote the distributions of amplitude, global phase, and polarization, respectively, varying along the predefined trajectory. $R\left(\cdot \right)=\left[\begin{array}{cc} \cos\left(\cdot \right) & \sin\left(\cdot \right)\\ -\sin\left(\cdot \right) & \cos\left(\cdot \right) \end{array}\right]$ denotes the rotation matrix. Additionally, the parameter $\rho =\frac{A_{R}\left(\xi \right)\sin\left(\Theta /2\right)}{A_{L}\left(\xi \right)\cos\left(\Theta /2\right)}$ is introduced to characterize the weighted amplitude ratio between the RCP and LCP components.

Under the prerequisite of a fixed incident polarization state (i.e., Θ and Φ remain constant), the parametric design of the dual‐channel complex amplitudes based on Equations (4), (5), (6), (7) enables independent and precise tuning of each DoF of the light field. Notably, whether these physical properties share common base components or not, they are completely decoupled in logic. Because the dual‐channel amplitude factors $A_{L}$ and $A_{R}$ are mutually independent, the trajectory amplitude A(ξ) and the weighted amplitude ratio ρ are fundamentally decoupled. Accordingly, while keeping the ratio ρ constant, scaling $A_{L}$ and $A_{R}$ simultaneously adjusts the intensity distribution along the trajectory; conversely, by actively regulating $A_{L}$, one can vary ρ (to modulate the ellipticity) while keeping the total amplitude A(ξ) constant. Furthermore, the global phase and polarization orientation angle, dictated by the topological charges $\left(l_{L},l_{R}\right)$ of the dual channels, are also decoupled. The global phase φ(ξ) is determined by the sum of the dual‐channel topological charges (i.e., the generalized phase order $l_{G}$), whereas the polarization orientation is explicitly controlled by their difference (i.e., the generalized polarization order $l_{P}$). This independent mathematical mapping ensures that by regulating the parameters of the dual‐channel complex amplitudes, fully structured and customizable VTBs can be achieved in the focal plane.

### 2.3 The Design of Metasurfaces

The realization of customizable fully structured VTBs demands that the metasurface be capable of independently manipulating the complex amplitudes of a pair of orthogonal polarization channels. The design of the metasurface is illustrated in Figure 2. We select anisotropic ${\text{TiO}}_{2}$ nanopillars exhibiting birefringence on a square $\text{Si}O_{2}$ substrate with a lattice constant of P=340 nm as a fundamental meta‐atom (Figure 2a) These structures possess a high refractive index and low loss across the visible spectrum [43]. Figure 2b displays the meta‐molecule structure composed of four meta‐atoms, where nanopillars A and B, fabricated from $\text{Ti}O_{2}$, are arranged alternately on the $\text{Si}O_{2}$ substrate. This structure is based on composite phase modulation and double‐atom interference mechanism, and can achieve the control of dual‐channel complex amplitude information [44, 45]. For the target reflection amplitudes $E_{L}=A_{L}e^{i\varphi_{L}}$ and $E_{R}=A_{R}e^{i\varphi_{R}}$ of the two channels, the transmission phase and rotation angles required for the nanopillars can be derived using the following relationship (see Supporting Information S1: Supplementary Section 2 for details):

(8) $$\begin{array}{c} \varphi_{x}^{A,B}=\frac{\varphi_{R}^{A,B}+\varphi_{L}^{A,B}}{2} \end{array},$$

(9) $$\begin{array}{c} \varphi_{y}^{A,B}=\varphi_{x}^{A,B}-\pi , \end{array}$$

(10) $$\begin{array}{c} \theta^{A,B}=\frac{\varphi_{R}^{A,B}-\varphi_{L}^{A,B}}{4} \end{array},$$

here $\varphi_{L}^{A}\left(x,y\right)=\varphi_{L}\left(x,y\right)+\cos^{-1}\left[A_{L}\left(x,y\right)\right]$, $\varphi_{L}^{B}\left(x,y\right)=\varphi_{L}\left(x,y\right)-\cos^{-1}\left[A_{L}\left(x,y\right)\right]$, $\varphi_{R}^{A}\left(x,y\right)=\varphi_{R}\left(x,y\right)+\cos^{-1}\left[A_{R}\left(x,y\right)\right]$, and $\varphi_{R}^{B}\left(x,y\right)=\varphi_{R}\left(x,y\right)-\cos^{-1}\left[A_{R}\left(x,y\right)\right]$ represent the four phases determined by $E_{L}$ and $E_{R}$, which respectively indicate the phase responses of nanopillars A and B to the two channels. Thus, by independently controlling these four parameters (the propagation and geometric phases for nanopillars A and B), we achieve complete and independent control over the amplitude and phase for each polarization channel.

**FIGURE 2:**
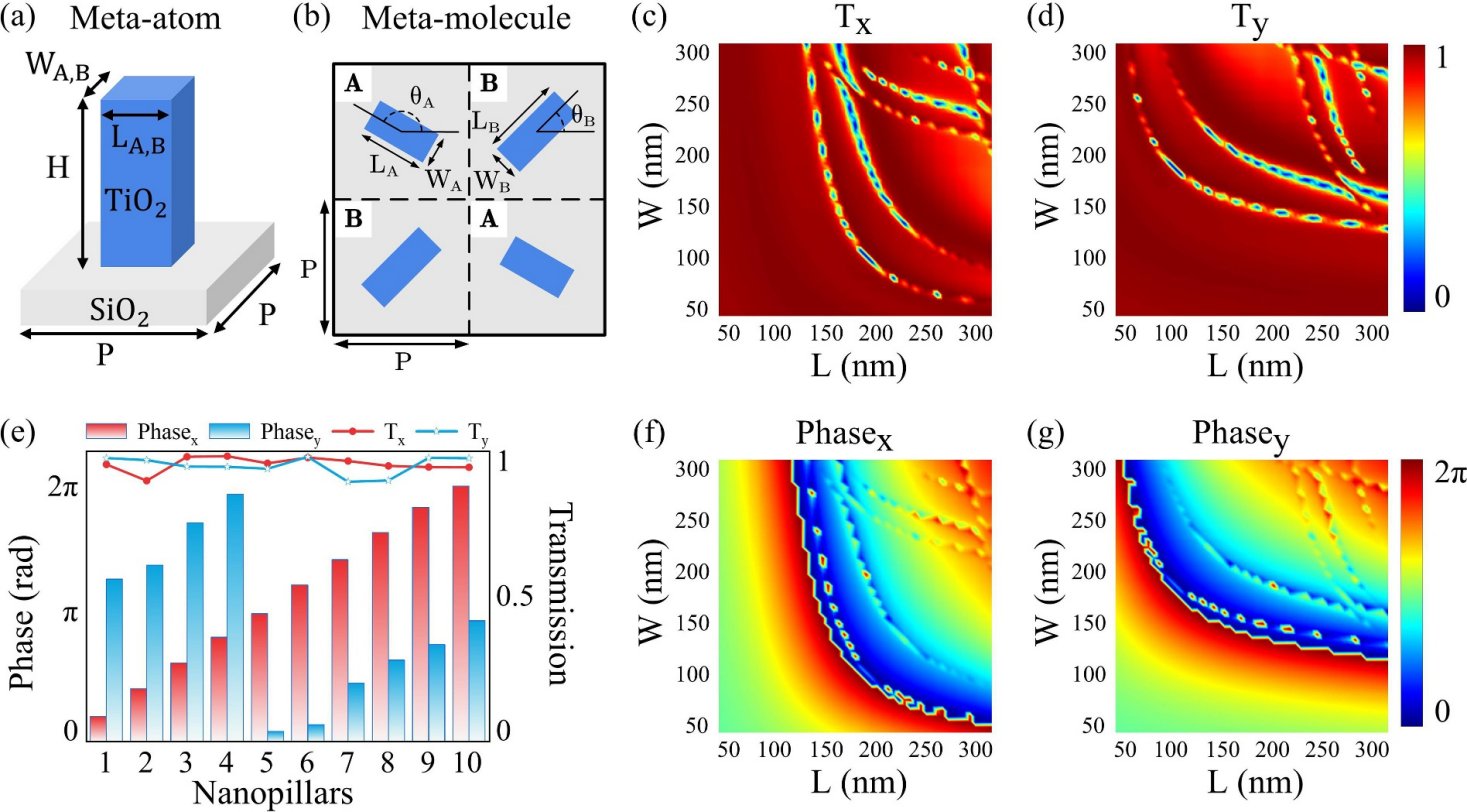
Schematic illustration of the metasurface design. (a) Perspective view of a meta‐atom of the dielectric metasurface. (b) Top view of the meta‐molecule. (c, f) Simulated transmission coefficients and phase shifts for *x*‐polarized incident light, plotted as functions of varying length and width. (d, g) Simulated transmission coefficients and phase shifts for *y*‐polarized incident light, plotted as functions of varying length and width. (e) Corresponding transmission coefficients and phase shifts of the 10 selected nanopillars.

Numerical simulations were performed to investigate the transmission characteristics of meta‐atoms with a fixed height of H=600 nm under normal incidence of a plane wave at λ=532 nm, utilizing the finite‐difference time‐domain (FDTD) method. In the simulations, periodic boundary conditions were applied along the *x* and *y* directions, whereas perfectly matched layers were imposed along the *z* direction. The simulated transmission coefficients and phase shifts are plotted as functions of the varying length and width of the nanopillars in Figure 2c,d,f,g. It is evident that by selecting appropriate combinations of lengths and widths, a full phase coverage of [0,2π] can be achieved. Figure 2e presents the transmittances and phase shifts of the 10 selected nanopillars. As the transmittances are close to 1, and the phase difference approaches π, each nanopillar functions as a highly efficient half‐wave plate. The detailed dimensions (lengths and widths) of these 10 nanopillars are listed in Supporting Information S1: Table S2 of the Supplementary Section 3. Based on these meta‐atoms, a metasurface with an aperture size of 44 μm × 44 μm was designed, comprising an 128×128 array of nanopillars with a target focal length of 80 μm. Regarding the numerical setup, an auto‐non‐uniform meshing algorithm was employed with the mesh accuracy set to 1. Based on the Rayleigh criterion, the theoretical trajectory width is calculated to be $w_{t}=0.61\lambda /\text{NA}=1.22$ μm.

## 3 Results and Discussion

### 3.1 Dual‐Channel Customizable Fully Structured TBs in the Focal Plane

To validate independent control over dual‐channel complex amplitudes, we implemented dual‐channel customizable fully structured TBs in the focal plane via a single‐layer metasurface (Figure 3). Following the theoretical design, the calculated amplitude and phase distributions required for the metasurface are illustrated in Supporting Information S1: Figure S1, with the corresponding spatial maps of the phase shifts and rotation angles for nanopillars A and B across the metasurface plane presented in Supporting Information S1: Figure S2 (see Supporting Information S1: Supplementary Section 4 for details). As shown in Figure 3a,b, when the metasurface is illuminated with LCP light and RCP light respectively, different effects will be observed in the focal plane. The corresponding results are presented in Figure 3c,d. In the LCP channel, a circular trajectory with a gradually increasing amplitude gradient is generated (the first column in Figure 3c). In the RCP channel, a hexagonal trajectory with alternating bright and dark amplitude distributions is presented (the first column in Figure 3d). This indicates that our method is capable of independently controlling the trajectory shapes of the LCP and RCP channels, as well as the amplitude variations along the preset trajectories. It is easily observed that the trajectory width is close to the theoretical value of 1.3 μm. The second column displays $S_{3}$, indicating that the spin state of the beam is reversed after modulation by the metasurface; this reflects the high purity of the channels and the high polarization conversion efficiency. The third column shows the preset topological structures in the phase distributions, where $l_{L}=4,l_{R}=6$. This indicates that the phases of the LCP and RCP channels can be precisely and independently controlled. The excellent agreement between FDTD simulations and the numerical calculations validates the proposed method.

**FIGURE 3:**
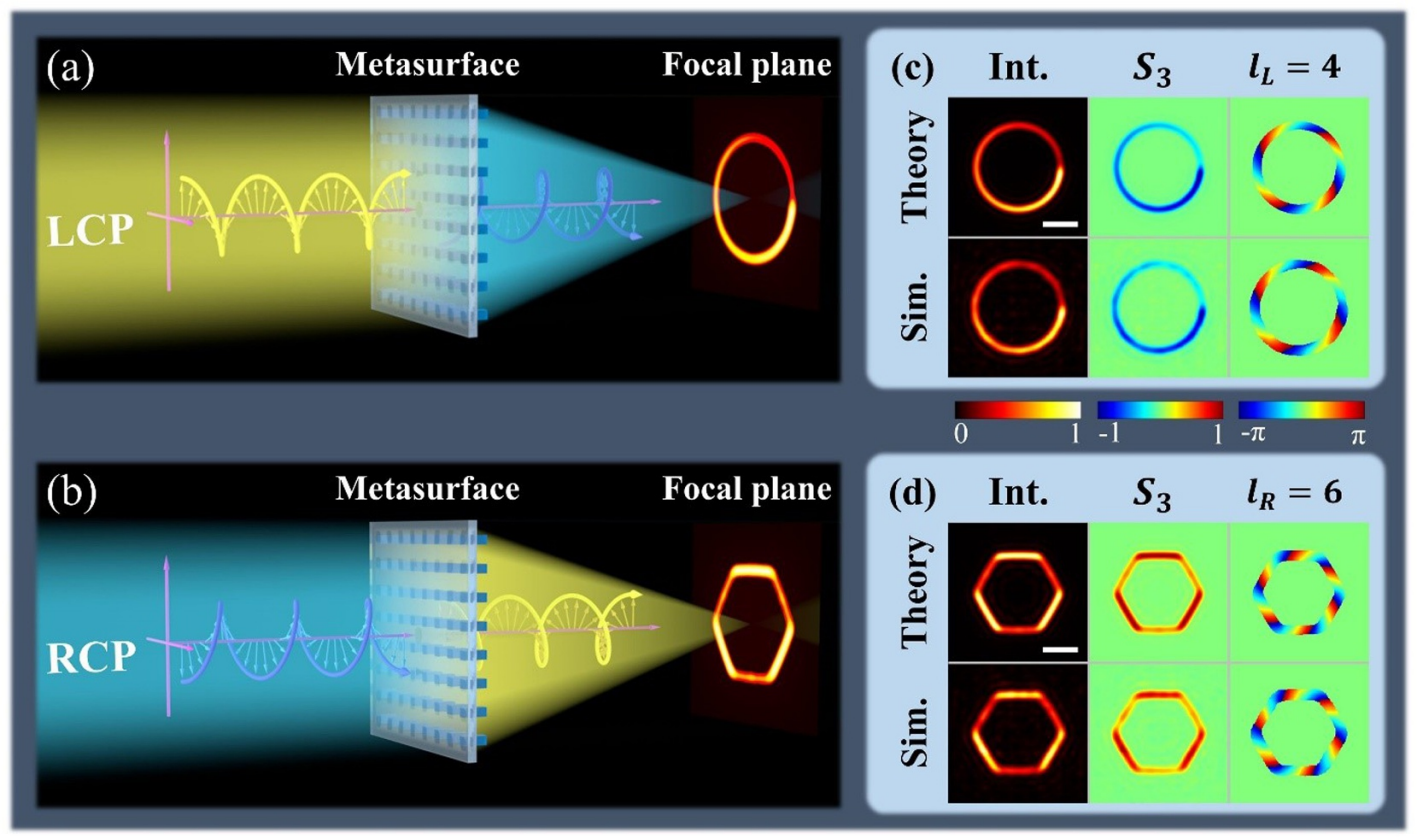
Dual‐channel customizable fully structured TBs in the focal plane. (a, b) Schematic illustrations under LCP and RCP incidence, respectively. (c, d) Distributions of intensity, Stokes parameters $S_{3}$, and phase in the focal plane under LCP and RCP incidence, respectively. The first and second rows correspond to theoretical calculations and FDTD simulation results, respectively. Scale bar: 5 μm.

In addition, the designed metasurface exhibits the expected broadband response characteristics. Supporting Information S1: Figure S3 of the Supplementary Section 5 illustrates the intensity and phase distributions of the TBs at various wavelengths, specifically 480, 532, 580, and 630 nm. At the corresponding focal planes of z=88,80,70,and66 μm, the in‐plane intensity patterns and phase orders maintain high similarity, demonstrating that the metasurface remains effective for generating TBs across a wide range of visible wavelengths. Furthermore, considering potential fabrication errors in actual processing, we calculated the focal‐plane field distributions when the nanopillar lengths and widths undergo deviations of ±10, ±20,±30,±40, and ±50 nm. As shown in Supporting Information S1: Figure S4 of the Supplementary Section 6, we numerically analyzed the focal‐plane field distributions under varying fabrication errors. The simulation results indicate that the amplitude and phase distributions of both channels maintain high fidelity for dimensional deviations within 30 nm. Although noticeable stray spots appear when the deviation reaches 50 nm, current fabrication techniques can generally be controlled within approximately 10 nm [46], further confirming the reliability of our design.

### 3.2 Customizable Fully Structured VTBs in the Focal Plane

The preceding section validated the metasurface’s capability for the independent manipulation of dual‐channel amplitude and phase. Therefore, by designing the metasurface to generate beams with identical trajectory shapes under both LCP and RCP illumination, the SoP distribution along the predefined trajectory can be tailored via the amplitude ratio and phase difference between these two orthogonal components.

In order to describe the characteristics of TBs, we compared and analyzed them with traditional Laguerre–Gaussian VVBs and GPVVBs that have the same phase order ($l_{G}$) and polarization order ($l_{P}$) as illustrated in Figure 4a. Conventional VVBs are typically restricted to circular trajectories. Specifically, for a high‐order VVB, the polarization orientation varies linearly only with the azimuthal coordinate (Figure 4a1), while a hybrid‐order VVB encompasses complex radial–azimuthal variations in both ellipticity and orientation (Figure 4a4). To overcome the limitation of fixed geometric profiles, high‐order and hybrid‐order GPVVBs have been introduced to enable customizable trajectory shapes, such as the “S”‐shaped GPVVBs demonstrated here (Figure 4a2,a5). However, the evolution of their light field parameters remains constrained by their intrinsic azimuthal dependence, leading to a mismatch between the polarization distribution and the trajectory shape. Furthermore, for both VVBs and GPVVBs, the presence of a central intensity null arising from phase singularities remains unavoidable (Figure 4a1,a2,a4,a5). In contrast, the VTBs proposed in this work overcome the limitations. For both high‐order (Figure 4a3) and hybrid‐order (Figure 4a6) configurations, the polarization distribution of VTBs evolves exclusively along the predefined trajectory, being solely determined by generalized polarization order ($l_{P}$). Finally, we demonstrate the normalized intensity distributions of an “S”‐shaped VTB after passing through analyzers oriented in various directions. The theoretical predictions show excellent agreement with the results obtained from FDTD simulations (Figure 4b).

**FIGURE 4:**
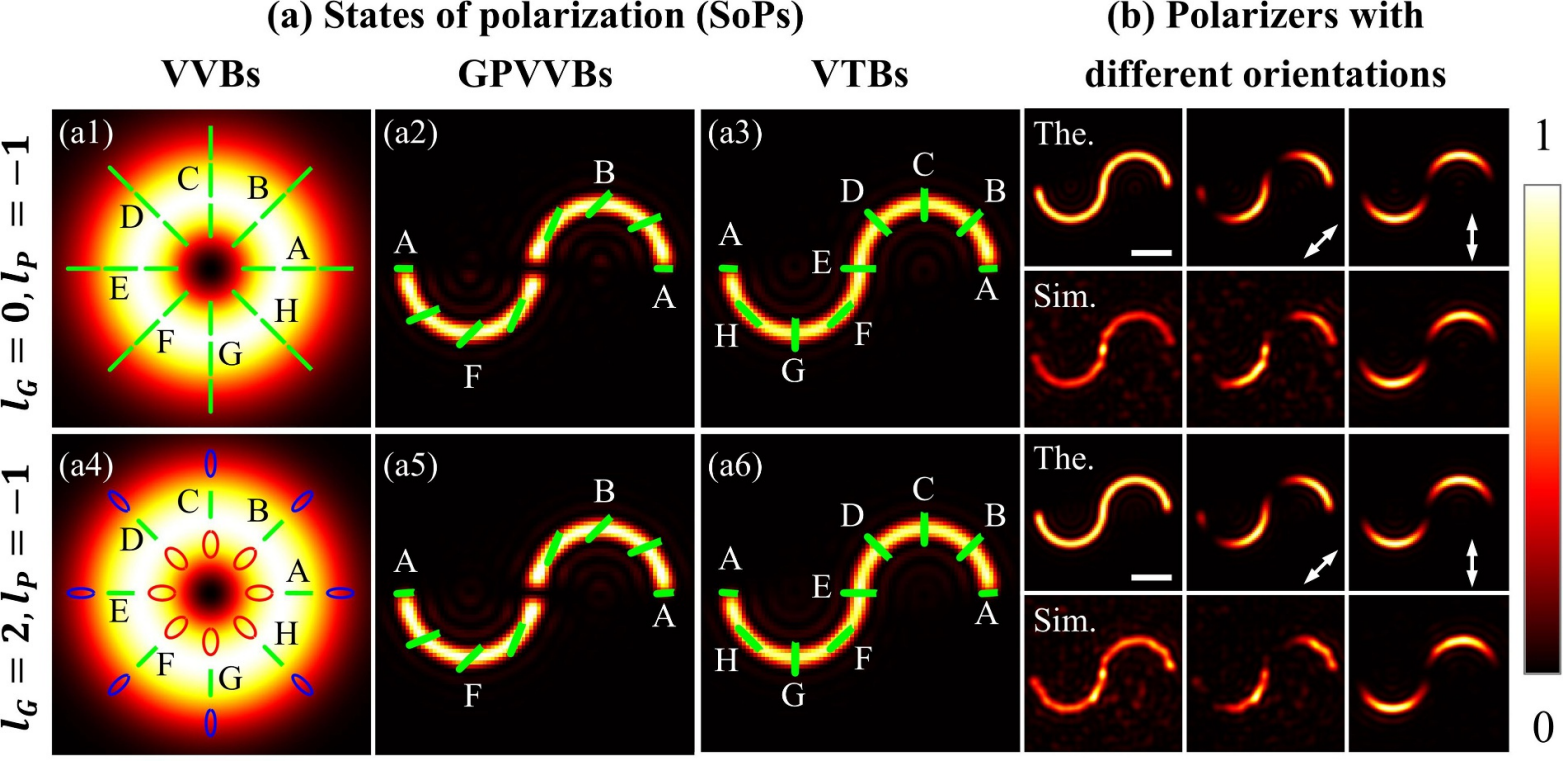
Comparison among conventional VVBs, GPVVBs and VTBs. (a) Polarization distributions of conventional VVBs (first column), GPVVBs (second column) and VTBs (third column). In each sub‐figure, the same letter labels (A–H) all correspond to the same SoPs. Green, red, and blue colors correspond to linear, left‐handed, and right‐handed polarization, respectively. (b) Normalized intensity distributions and intensity patterns after passing through analyzers with different orientations for the generated high‐order and hybrid‐order VTBs. The first and third rows correspond to theoretical calculations, while the second and fourth rows display FDTD simulation results. The white double‐headed arrows denote the analyzer direction. Scale bar: 5 μm.

Beyond the trajectory‐dependent evolution of field parameters, the core advantage of the proposed VTBs lies in the decoupling of multiple DoFs, specifically evidenced by the generalized phase order ($l_{G}$) and the generalized polarization order ($l_{P}$). Under illumination with a fixed SoP $\left(\Theta ,\Phi \right)=\left(\frac{\pi }{2},0\right)$, we analyzed VTBs with identical generalized polarization orders ($l_{P}$) but different generalized phase orders ($l_{G}$), as well as those with different generalized polarization orders ($l_{P}$) but identical generalized phase orders ($l_{G}$). Figure 5 presents the results obtained from theoretical calculations and FDTD simulations, showing the intensity distributions under analyzers oriented at different angles (0,π/4,π/2,3π/4), along with the extracted phase distributions for the LCP and RCP components, as well as the global phase distributions. Comparing Figure 5a $\left(l_{G}=3,l_{P}=-1\right)$ and Figure 5b $\left(l_{G}=4,l_{P}=-1\right)$, it is evident that VTBs can carry different global phases along the predefined trajectory while maintaining the same SoPs. Conversely, a comparison of Figure 5b $\left(l_{G}=4,l_{P}=-1\right)$ and Figure 5c $\left(l_{G}=4,l_{P}=-2\right)$ reveals that VTBs with identical global phases can exhibit distinct polarization distributions along the predefined trajectory. These findings further confirm the complete decoupling of the polarization and phase orders of VTBs designed by the proposed method. Although consistent with results reported [24], our work distinguishes itself by extending the concept to arbitrary shapes, with a specific focus on TBs rather than azimuthally varying vortex beams.

**FIGURE 5:**
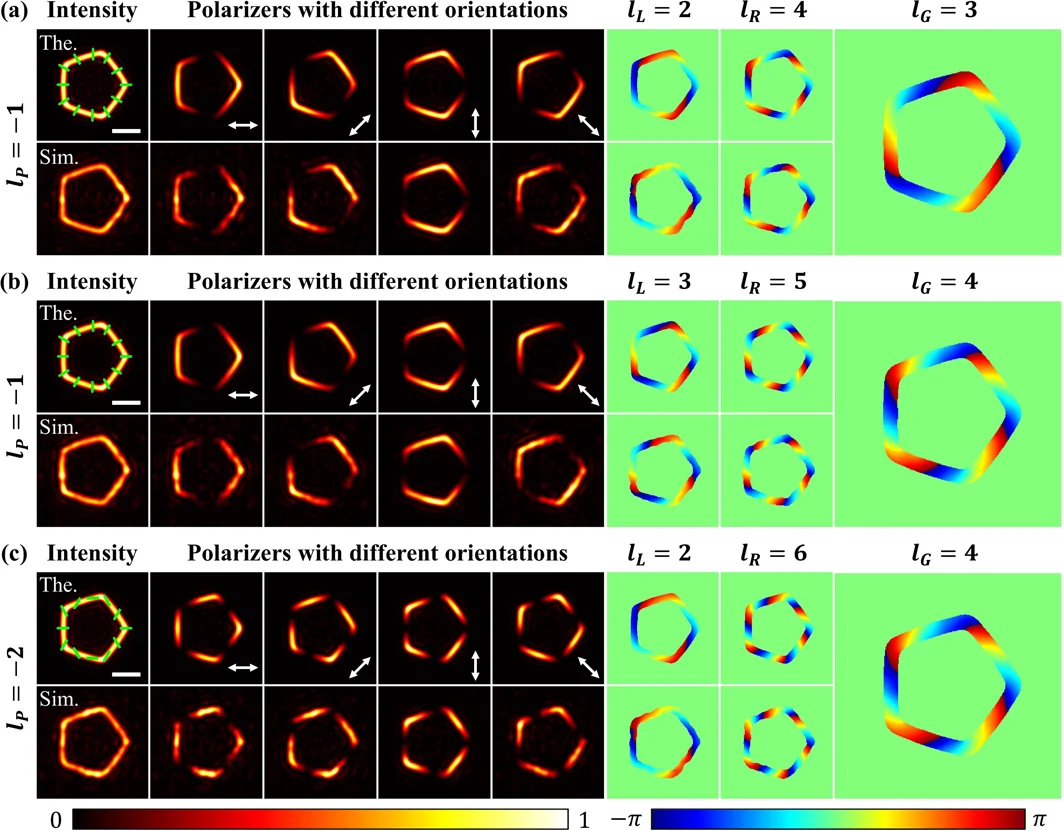
Investigation of the generalized phase and polarization orders of VTBs. (a) $l_{G}=3,l_{P}=-1$. (b) $l_{G}=4,l_{P}=-1$. (c) $l_{G}=4,l_{P}=-2$. The panels display the normalized intensity distributions after passing through analyzers with different orientations, the extracted phase distributions of the LCP and RCP components, and the global phase distributions for the VTBs. These results are obtained from both theoretical calculations and FDTD simulations. Green color corresponds to linear polarization. And the white double‐headed arrows denote the analyzer direction. Scale bar: 5 μm.

Building upon the successful decoupling of generalized phase order ($l_{G}$) and polarization order ($l_{P}$), we further achieved the modulation of ellipticity and initial orientation angle by varying the incident polarization state. For a more systematic description, we introduce the ${\text{PS}}^{\left(l_{G},l_{P}\right)}$, which denotes the Poincaré sphere, with the superscripts representing the generalized phase order ($l_{G}$) and polarization order ($l_{P}$), respectively. Figure 6 illustrates the generated VTBs on ${\text{PS}}^{\left(0,-2\right)}$ under arbitrary incident SoPs on the fundamental PS, hereafter referred to as ${\text{PS}}^{\left(0,0\right)}$. As representative examples, we highlight several cases described by coordinates of $A\left(\frac{\pi }{4},0\right),B\left(\frac{\pi }{2},0\right),C\left(\frac{\pi }{2},\frac{\pi }{2}\right),D\left(\frac{3\pi }{4},\frac{\pi }{2}\right)$ on ${\text{PS}}^{\left(0,0\right)}$, including their schematic polarization patterns (Figure 6a1–d1) and the corresponding Stokes parameter distributions. Notably, the incident polarization and the generated output correspond to identical coordinates on their respective PS. Accordingly, $S_{0}$ consistently exhibits the predefined pentagon trajectory, with the polarization distribution evolving along the predefined trajectory. The states on the equator are characterized by pronounced linear polarization (where $S_{1}$ and $S_{2}$ at points B and C appear brighter compared to points A and D), and the circular polarization component approaches 0 ($S_{3}$ at points B and C). The northern hemisphere exhibits left‐handed elliptical polarization ($S_{3}>0$), whereas the southern hemisphere exhibits right‐handed elliptical polarization ($S_{3}<0$). The excellent agreement between the theoretical calculations (first row) and FDTD simulation results (second row) validates the effectiveness of the proposed method.

**FIGURE 6:**
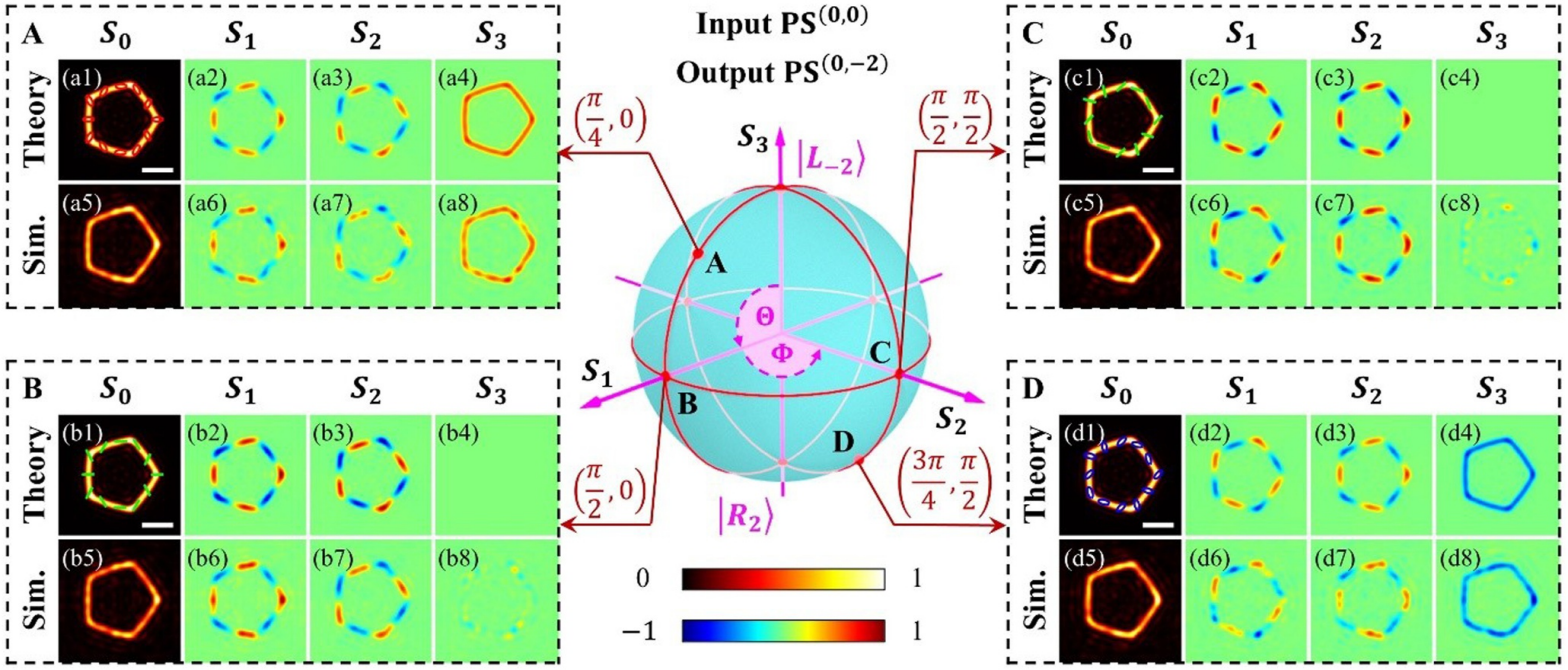
VTBs on the ${\text{PS}}^{\left(0,-2\right)}$. (A–D) Pentagon VTBs with distinct polarization distributions on ${\text{PS}}^{\left(0,-2\right)}$ and their corresponding Stokes parameter distributions under various incident SoPs $A\left(\frac{\pi }{4},0\right),B\left(\frac{\pi }{2},0\right),C\left(\frac{\pi }{2},\frac{\pi }{2}\right),D\left(\frac{3\pi }{4},\frac{\pi }{2}\right)$ on ${\text{PS}}^{\left(0,0\right)}$, respectively. In each panel, the first and second rows display the theoretical calculations and simulation results, respectively. Green, red, and blue colors correspond to linear, left‐handed, and right‐handed polarization, respectively. Scale bar: 5 μm.

Finally, to validate the capability of the proposed method in generating customizable fully structured VTBs under specific incident polarization light, we designed a single‐layer metasurface capable of generating a VTB with a distinctive spatial structure under *x*‐polarized illumination. Specifically, the trajectory shape is designed as a “lips”‐shaped. The amplitude distribution is characterized by a dimmer upper half and a brighter lower half while carrying a global phase of the third order. The polarization distribution evolves along two distinct paths on ${\text{PS}}^{\left(0,0\right)}$. Figure 7a illustrates the polarization design concept: starting from point $A\left(\frac{\pi }{2},0\right)$ on ${\text{PS}}^{\left(0,0\right)}$, the state first completes one full rotation along the path $C_{1}$ returning to point A, and subsequently completes another rotation along the path $C_{2}$ back to point A. The specific polarization evolution along these two paths is depicted in Figure 7b. Figure 7d presents the results for intensity and phase. The intensity distribution exhibits the “lips”‐shaped trajectory with the designed “dim‐top and bright‐bottom” contrast. By extracting the phase distributions of the LCP and RCP components of the total field, a value of $\left(l_{L}=2,l_{R}=4\right)$ is obtained, indicating a global vortex phase of the third order $\left(l_{G}=3\right)$. The polarization distribution is displayed in Figure 7e; as intended, the SoPs along the trajectory follow the evolution along the two paths $C_{1}$ and $C_{2}$ on the ${\text{PS}}^{\left(0,0\right)}$, with specific Stokes parameter distributions provided. To further demonstrate the flexibility of our method in generating arbitrary beams, Figure 7f,g display another VTB with the same amplitude distributions as those in Figure 7d,e, but with different phase orders $\left(l_{G}=4\right)$ and polarization distributions. Specifically, the evolution of their SoPs along the trajectory completes four full cycles around path $C_{1}$ on the ${\text{PS}}^{\left(0,0\right)}$. Furthermore, the results obtained from the FDTD method demonstrate excellent agreement with the theoretical calculations. This agreement confirms that our method successfully achieves independent control over the four key DoFs—trajectory shape and the distributions of amplitude, phase, and polarization along the predefined trajectory—enabling the generation of customizable fully structured VTBs in the focal plane via a single‐layer metasurface.

**FIGURE 7:**
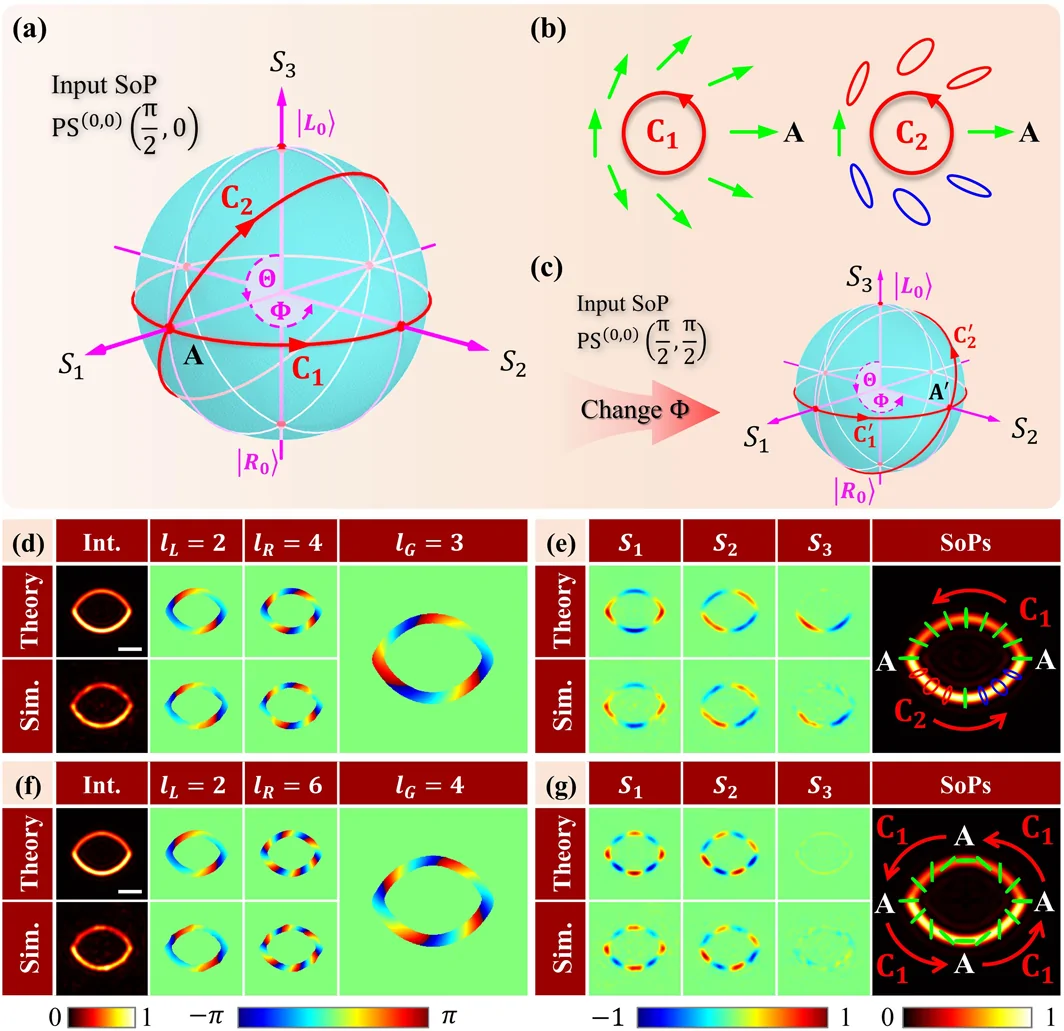
A customizable fully structured VTB in the focal plane. (a) Visualization of the SoP paths $C_{1}$ and $C_{2}$ on ${\text{PS}}^{\left(0,0\right)}$ under incident SoP $\left(\frac{\pi }{2},0\right)$ on ${\text{PS}}^{\left(0,0\right)}$. (b) Specific polarization evolution along paths $C_{1}$ and $C_{2}$. (c) Visualization of the SoP paths $C_{1}^{\prime }$ and $C_{2}^{\prime }$ on ${\text{PS}}^{\left(0,0\right)}$ under incident SoP $\left(\frac{\pi }{2},\frac{\pi }{2}\right)$ on ${\text{PS}}^{\left(0,0\right)}$. (d) Intensity and phase distributions obtained from theoretical calculations and FDTD simulations. (e) Stokes parameters and polarization distributions along the predefined trajectory obtained from theoretical calculations and FDTD simulations. (f, g) VTB with identical amplitude distributions but distinct phase orders and SoPs. Green, red, and blue colors correspond to linear, left‐handed, and right‐handed polarization, respectively. Scale bar: 5 μm.

As indicated by Equations ((4), (5), (6), (7)), the global control factor Φ enables independent manipulation of the polarization orientation angle without perturbing the amplitude and phase distributions. As illustrated in Figure 7c, when the incident polarization is switched from $\left(\frac{\pi }{2},0\right)$ to $\left(\frac{\pi }{2},\frac{\pi }{2}\right)$ on ${\text{PS}}^{\left(0,0\right)}$, we observe only a global rotation of the polarization orientation angle by $\frac{\pi }{4}$. Specifically, the polarization evolution proceeds as follows: Starting from point $A^{\prime }\left(\frac{\pi }{2},\frac{\pi }{2}\right)$, the state traverses one full loop along path $C_{1}^{\prime }$ to return to $A^{\prime }$, and subsequently follows path $C_{2}^{\prime }$ back to $A^{\prime }$. Detailed Stokes parameter distributions are provided in Supporting Information S1: Figure S5 (see Supporting Information S1: Supplementary Section 7 for details). Compared with the VTBs on ${\text{PS}}^{\left(0,-2\right)}$ shown in Figure 6 and the conventional PS beams, the SoP along the trajectory designed by us can have arbitrary ellipticity and orientation angle. This capability significantly extends the DoFs and flexibility in polarization control compared to traditional schemes. Such dynamic and independent control offers a new dimension for constructing customized optical fields, demonstrating broad potential applications in fields such as dynamic encryption and secure communication.

## 4 Conclusion

In conclusion, we have systematically proposed and demonstrated a method for generating TBs via a single‐layer all‐dielectric metasurface. By integrating computer‐generated holography with composite phase modulation and a diatomic interference mechanism, on‐demand customizable fully structured VTBs in the focal plane—including the trajectory shape and the distributions of amplitude, phase, and polarization along the predefined trajectory—can be achieved. Compared with previous studies, this method not only overcomes the restriction that the vortex phase must strictly vary along the azimuthal direction but also significantly enhances the DoFs for independent control. As a proof of concept, we first demonstrated the generation of dual‐channel customizable fully structured TBs in the focal plane via a single‐layer metasurface, where the trajectory shape and the distributions of intensity and phase for each channel were independently controlled. Furthermore, via the coherent superposition of two orthogonal channels, we realized a “lips”‐shaped fully structured VTB with completely decoupled spatial structural parameters under specific incident polarization illumination. This successfully validates the independent control over the trajectory shape and the distributions of amplitude, phase, and polarization along the predefined trajectory. This research provides an efficient and flexible technical pathway for the advanced tailoring of complex structured light fields. This work unlocks significant potential for advanced optical applications, such as enhancing information capacity and transmission speed in optical communications [19, 29, 30, 47, 48, 49, 50], and facilitating the exploration of light–matter interaction mechanisms in particle manipulation by leveraging multiple DoFs that vary along the trajectory.

## Author Contributions

**Zhibang Tan:** conceptualization, investigation, methodology, validation, visualization, writing – original draft, software, writing – review and editing. **Zebin Chen:** writing – review and editing, software. **Zepeng Chen:** writing – review and editing, software. **Yantao Zheng:** software, writing – review and editing. **Min Ouyang:** supervision, writing – review and editing. **Wei Hu:** funding acquisition, resources, supervision. **Qing Wang:** funding acquisition, resources, supervision. **Lijun Wu:** funding acquisition, resources, supervision. **Qian Shou:** funding acquisition, investigation, conceptualization, visualization, validation, methodology, writing – review and editing, supervision.

## Supporting information


Supporting Information S1

## Data Availability

The data that support the findings of this study are available from the corresponding author upon reasonable request.
