# Supplementary material for: Customizable Fully Structured Vector Trajectory Beams in the Focal Plane via a Single‐Layer Metasurface
Source: Nanophotonics. 2026 May 24;15(10):e70141. doi: 10.1002/nap2.70141 (PMC13642520; doi:10.1002/nap2.70141)
Supplement: Supplementary file 1 [file NAP2-15-e70141-s001.docx]

Supporting Information

Customizable Fully Structured Vector Trajectory Beams in the Focal Plane via a Single-Layer Metasurface

*Zhibang Tan, Zebin Chen, Zepeng Chen, Yantao Zheng, Min Ouyang, Wei Hu, Qing Wang, Lijun Wu, and Qian Shou**

**Supplementary Section 1. Comparison with Related Works**

**Supplementary Section 2. Design Principle of the Metasurface**

**Supplementary Section 3. Geometrical Parameters of Nanopillars**

**Supplementary Section 4. Theoretical Design Requirements and Structural Mapping of the Metasurface**

**Supplementary Section 5. Broadband Response and Focal-Plane Intensity and Phase Analysis**

**Supplementary Section 6. Numerical Analysis of Fabrication Errors**

**Supplementary Section 7. Polarization Analysis under Incident SoP** $\left( \frac{\boldsymbol{\pi}}{\boldsymbol{2}}\boldsymbol{,}\frac{\boldsymbol{\pi}}{\boldsymbol{2}} \right)$ **on** $\mathbf{PS}^{\left( \mathbf{0,0} \right)}$

**Supplementary Section 1.** **Comparison with Related Works**

Here, the table header is explained in detail as follows: Shape: Refers to whether the trajectory shape can be freely customized. Trajectory (Tra.): Refers to whether the DoFs of the light field are distributed along the preset trajectory. Amplitude (Amp.): Refers to whether the amplitude distribution of the light field can be independently regulated. Phase: Refers to the ability to independently control the phase of two orthogonal channels that constitute a vector beam. Orientation Angle (Ori. Angle) and Ellipticity (Ell.): Refers to whether the polarization of the light field can be independently adjusted. Perfect: Refers to whether the beam size is independent of the topological charge. Broadband (Bro.): Refers to whether metasurface possesses a broadband response. Efficiency (Eff.): The efficiency of the designed metasurface.

**Table S1. Comparison between this work and related works that only use metasurfaces.**

| Works | Year | Shape | Tra. | Amp. | Phase | Ori. Angle | Ell. | Perfect | Bro. | Eff. |
| --- | --- | --- | --- | --- | --- | --- | --- | --- | --- | --- |
| ^[24]^ | 2020 |  |  |  | √ | √ | √ | √ |  | 18% |
| ^[28]^ | 2024 | √ |  |  | √ | √ |  | √ | √ | 85% |
| ^[27]^ | 2025 | √ |  |  |  | √ |  | √ |  |  |
| ^[19]^ | 2025 |  |  | √ | √ | √ | √ | √ |  |  |
| ^[37]^ | 2025 | √ | √ |  | √ | √ |  | √ | √ | 70% |
| This work |  | √ | √ | √ | √ | √ | √ | √ | √ | 30% |

**Supplementary Section 2. Design Principle of the Metasurface**

For the complex amplitudes $E_{L}$ and $E_{R}$ of the two Customizable Fully Structured TBs in the focal plane obtained in Section 2.1, the required Jones matrix $J$ of the metasurface must satisfy:

$$\begin{aligned} J\left( x,y \right)\left| \left. L \right\rangle\right.=E_{R}\left( x,y \right)\left| \left. R \right\rangle\right.=A_{R}\left( x,y \right)e^{i\varphi_{R}\left( x,y \right)}\left| \left. R \right\rangle\right.\#\left( S1 \right) \end{aligned}$$

$$\begin{aligned} J\left( x,y \right)\left| \left. R \right\rangle\right.=E_{L}\left( x,y \right)\left| \left. L \right\rangle\right.=A_{L}\left( x,y \right)e^{i\varphi_{L}\left( x,y \right)}\left| \left. L \right\rangle\right.\#\left( S2 \right) \end{aligned}$$

Here, $A_{R}\left( x,y \right)e^{i\varphi_{R}\left( x,y \right)}$ and $A_{L}\left( x,y \right)e^{i\varphi_{L}\left( x,y \right)}$ represent the target complex amplitudes for the two channels. Solving for these conditions, the required Jones matrix of the metasurface is derived as:

$$\begin{aligned} J=\frac{1}{2}\left( \begin{matrix} E_{R}\left( x,y \right)+E_{L}\left( x,y \right) & -i\left( E_{R}\left( x,y \right)-E_{L}\left( x,y \right) \right) \\ -i\left( E_{R}\left( x,y \right)-E_{L}\left( x,y \right) \right) & -\left( E_{R}\left( x,y \right)+E_{L}\left( x,y \right) \right) \end{matrix} \right)\#\left( S3 \right) \end{aligned}$$

Mathematically, the target complex amplitude can be decomposed into a linear combination of two pure phase terms^[44,45]^:

$$\begin{aligned} A_{L,R}\left( x,y \right)e^{i\varphi_{L,R}\left( x,y \right)}=\cos\left( \frac{\varphi_{L,R}^{A}-\varphi_{L,R}^{B}}{2} \right)e^{i\left( \frac{\varphi_{L,R}^{A}+\varphi_{L,R}^{B}}{2} \right)}=\frac{\left( e^{i\varphi_{L,R}^{A}}+e^{i\varphi_{L,R}^{B}} \right)}{2}\#\left( S4 \right) \end{aligned}$$

Here, $\varphi_{L}^{A}\left( x,y \right)={\varphi_{L}\left( x,y \right)+\cos}^{-1} \left[ A_{L}\left( x,y \right) \right]$, $\varphi_{L}^{B}\left( x,y \right)=\varphi_{L}\left( x,y \right)-\cos^{-1} \left[ A_{L}\left( x,y \right) \right]$, $\varphi_{R}^{A}\left( x,y \right)={\varphi_{R}\left( x,y \right)+\cos}^{-1} \left[ A_{R}\left( x,y \right) \right]$, and $\varphi_{R}^{B}\left( x,y \right)=\varphi_{R}\left( x,y \right)-\cos^{-1} \left[ A_{R}\left( x,y \right) \right]$. Substituting Eq. (S4) into Eq. (S3) yields:

$$\begin{aligned} J\left( x,y \right)=\frac{1}{2}\left[ J^{A}\left( x,y \right)+J^{B}\left( x,y \right) \right]\#\left( S5 \right) \end{aligned}$$

The mentioned derivation successfully decomposes the target complex amplitude into a linear combination of two phase factors. To implement this design via a metasurface, it is essential to establish a mapping between the nanostructural parameters and these phase factors. Anisotropic nanopillars $A$ and $B$ exhibit distinct electromagnetic responses along their major and minor axes; their optical response, when aligned with the metasurface $x$-$y$ coordinate system, is described by $J^{A,B}=\left[ \begin{matrix} t_{x}^{A,B} & 0 \\ 0 & t_{y}^{A,B} \end{matrix} \right]$. Upon rotating the nanostructure by an angle $\theta$ relative to the metasurface coordinate system, the resulting Jones matrix can be expressed as:

$$\begin{aligned} J_{\theta}^{A,B}=R\left( -\theta^{A,B} \right)\left[ \begin{matrix} t_{x}^{A,B} & 0 \\ 0 & t_{y}^{A,B} \end{matrix} \right]R\left( \theta^{A,B} \right)\#\left( S6 \right) \end{aligned}$$

Here, $t_{x}^{A,B}=T_{x}^{A,B}e^{i\varphi_{x}^{A,B}}$ and $t_{y}^{A,B}=T_{y}^{A,B}e^{i\varphi_{y}^{A,B}}$ denote the amplitude transmission coefficients and phase retardations of the anisotropic structures $A$ and $B$ along the $x$ and $y$ polarization directions, respectively. When the transmittance approaches unity, Eq. (S6) can be expressed as:

$$\begin{aligned} J^{A,B}=\left[ \begin{matrix} e^{i\varphi_{x}^{A,B}}\cos^{2}\theta+e^{i\varphi_{y}^{A,B}}\sin^{2}\theta& \left( e^{i\varphi_{x}^{A,B}}-e^{i\varphi_{y}^{A,B}} \right)\sin\theta\cos\theta\\ \left( e^{i\varphi_{x}^{A,B}}-e^{i\varphi_{y}^{A,B}} \right)\sin\theta\cos\theta& e^{i\varphi_{x}^{A,B}}\sin^{2}\theta+e^{i\varphi_{y}^{A,B}}\cos^{2}\theta\end{matrix} \right]\#\left( S7 \right) \end{aligned}$$

By equating the terms $J^{A}\left( x,y \right)$ and $J^{B}\left( x,y \right)$ in Eq. (S5) with those in Eq. (S7) and solving the resulting system of equations, we obtain:

$$\begin{aligned} \varphi_{x}^{A,B}=\frac{\varphi_{R}^{A,B}+\varphi_{L}^{A,B}}{2}\#\left( S8 \right) \end{aligned}$$

$$\begin{aligned} \varphi_{y}^{A,B}=\varphi_{x}^{A,B}-\pi\#\left( S9 \right) \end{aligned}$$

$$\begin{aligned} \theta^{A,B}=\frac{\varphi_{R}^{A,B}-\varphi_{L}^{A,B}}{4}\#\left( S10 \right) \end{aligned}$$

Here, $\varphi_{x}^{A,B}、\varphi_{y}^{A,B}、\theta^{A,B}$ denote the propagation phases and geometric phases of nanopillars A and B along their major and minor axes, respectively. Consequently, the simultaneous manipulation of the geometric and propagation phases of the two anisotropic nanopillars A and B enables the independent control of amplitude and phase for a pair of orthogonal circular polarization channels.

**Supplementary Section 3. Geometrical Parameters of Nanopillars**

**Table S2. The geometrical parameters of the ten nanopillars selected to cover the entire** $\boldsymbol{2}\boldsymbol{\pi}$ **phase range.**

| Nanopillars | Length (nm) | Width (nm) | Nanopillars | Length (nm) | Width (nm) |
| --- | --- | --- | --- | --- | --- |
| 1 | 70 | 285 | 6 | 295 | 65 |
| 2 | 80 | 290 | 7 | 285 | 85 |
| 3 | 105 | 270 | 8 | 275 | 100 |
| 4 | 115 | 280 | 9 | 280 | 110 |
| 5 | 120 | 300 | 10 | 300 | 115 |

**Supplementary Section 4. Theoretical Design Requirements and Structural Mapping of the Metasurface**

**Figure S1**. Calculated target complex amplitude distributions for the dual-channel metasurface. (a, b) Spatial distributions of the required amplitude and (c, d) the corresponding phase profiles for the LCP and RCP channels, respectively, designed to generate the customizable fully structured TBs. These distributions serve as the theoretical basis for determining the local meta-atom parameters.

**Figure S2**. The corresponding phase shifts $\left( \varphi_{Ax},\varphi_{Ay},\varphi_{Bx},\varphi_{By} \right)$ and rotation angles $\left( \theta_{A},\theta_{B} \right)$ of nanopillars A and B , mapped as a function of the spatial coordinates in the metasurface plane.

**Supplementary Section 5. Broadband Response and Focal-Plane Intensity and Phase Analysis**

**Figure S3**. Broadband response of the metasurface. Simulated intensity distributions and phase distributions of the TBs at incident wavelengths of 480 nm, 532 nm, 580 nm, and 630 nm, respectively. The profiles are captured at the corresponding focal planes of $z=88 \mu m, 80 \mu m, 70 \mu m, and 66 \mu m$.

**Supplementary Section 6. Numerical Analysis of Fabrication Errors**

**Figure S4**. Numerical simulation of focal-plane field distributions under varying fabrication errors.Simulated amplitude and phase distributions of the LCP and RCP channels in the focal plane, considering dimensional deviations in nanopillar lengths and widths of $\pm10 \mathrm{nm}$, $\pm20 \mathrm{nm}, \pm30 \mathrm{nm}, \pm40 \mathrm{nm},$ and $\pm50 \mathrm{nm}$.

**Supplementary Section 7. Polarization Analysis under Incident SoP** $\left( \frac{\boldsymbol{\pi}}{\boldsymbol{2}}\boldsymbol{,}\frac{\boldsymbol{\pi}}{\boldsymbol{2}} \right)$ **on** $\mathbf{PS}^{\left( \mathbf{0,0} \right)}$

**Figure S5**. The distribution of SoPs and the corresponding distribution of Stokes parameters (the first row shows the theoretical results, the second row shows the FDTD simulation results) under incident SoP $\left( \frac{\pi}{2},\frac{\pi}{2} \right)$ on $\mathrm{PS}^{\left( 0,0 \right)}$.
